# Replication of central policy or local innovation: a study of the policy reinvention mechanism for sports policies for older adults in China’s provincial local governments

**DOI:** 10.3389/fpubh.2026.1820502

**Published:** 2026-05-29

**Authors:** Fangyang Zhang, Ke Zhou, Xiaoqiang Shi

**Affiliations:** School of Economics and Management, Shanghai University of Sport, Shanghai, China

**Keywords:** China, local innovation, policy reinvention, provincial local governments, sports policy for older adults

## Abstract

**Objective:**

This study examines how Chinese provincial governments adapt central sports policies for older adults. It aims to measure the degree of local policy reinvention and to identify the combinations of conditions under which substantive local redesign is more likely to occur.

**Methods:**

A total of 109 central and provincial policy documents on sports for older adults issued between 2015 and 2024 were collected and systematically analyzed. First, a cosine similarity method based on TF-IDF is used to compare the one-to-one matching central and provincial policy texts to construct a policy reinvention coefficient. Second, fsQCA was applied in a periodized design aligned with the 13th and 14th Five-Year Plan stages to examine how per capita provincial-level sports fiscal expenditure, population aging, public sports service resources, policy salience in aging planning, horizontal pressure, and adoption timing jointly shape high reinvention outcomes within each planning stage.

**Results:**

The overall level of policy reinvention is relatively high, with clear provincial variation but no extreme polarization. Reinvention fluctuated in the earlier years and became more stable after 2020. The periodized fsQCA further shows that the mechanisms of high reinvention differ across planning stages. In the 13th Five-Year Plan stage, five configurations were identified and grouped into delayed learning-adjustment, rapid capacity-response, and constraint-compensating types. In the observed 14th Five-Year Plan stage, seven configurations were identified and grouped into fiscal-demand, horizontal-learning, and aging-response types. These results suggest that provincial governments redesign central directives through stage-specific combinations of demographic demand, sports sector fiscal support, service resources, policy attention, adoption timing, and interprovincial learning.

**Conclusion:**

The local adaptation of sports policies for older adults in China follows multiple pathways rather than a single uniform model. Policy reinvention is shaped by both stable provincial conditions and stage-specific governance contexts. The periodized results show that early-stage reinvention relied more on learning, adjustment time, and resource preparation, while later stage reinvention was more strongly associated with aging pressure, sports sector fiscal support, and horizontal policy learning. These findings contribute to research on sports policy for older adults and offer evidence for improving the local implementation of healthy aging policies.

## 1 Introduction

Sports policy has become an increasingly important instrument for promoting healthy aging and social participation. Population aging is a major global challenge and a trend that is universal and irreversible (1). For the growing older population, such policies are especially important. They help promote active aging and improve quality of life. In China, the active response to population aging has become a national strategy, and sports policy for older adults now links healthy aging, national fitness, and community public service provision. This broader policy significance is well documented in research on population aging, healthy aging, and older-adult sport participation (2–5).

In this context, the central government provides the basic policy framework for sports for older adults, and provincial governments are responsible for translating broad directives into locally workable plans, implementation schemes, and operational rules. During the upward transmission process, local governments need to refine and update their central policy texts, which is a process known as policy reinvention (6). Through policy reinvention, local governments can integrate specific local conditions, such as regional aging characteristics, public sports facility conditions, and the actual exercise needs of older adults, to better adapt national macro policies to local realities, thereby enhancing policy relevance and operability.

However, in the actual policy transmission process, some local governments merely copy and forward documents, acting only as a policy transmitters without providing the local refinement and substantive innovation that policies require (7). This relatively low degree of policy reinvention can easily lead to the implementation of unsuitable policies, thereby influencing the effectiveness and execution quality of national sports policies for older adults. The reasons for this may stem from insufficient attention given by local governments to sports for older adults, limited policy execution capacity, inaccurate understanding of local aging characteristics, etc.

This study therefore addresses one main question: under what combinations of the needs of older adults, per capita provincial-level sports fiscal expenditure, public sports service resources, policy salience in aging planning, horizontal pressure, and adoption timing do Chinese provinces substantially reinvent central sports policies for older adults? Two sub-questions further specify this inquiry. First, how does the degree of policy reinvention vary across provinces and over time? Second, which causal configurations are associated with high levels of reinvention? To answer these questions, we combine text similarity analysis with fsQCA and examine policy documents issued by 31 provincial governments. The findings are expected to advance understanding of vertical policy adaptation and provide evidence for improving the local implementation of sports policies for older adults.

This article contributes to the literature on sports policy for older adults in three respects. First, it moves beyond the question of whether local governments adopt central policies and examines how they adapt policy content after adoption. In doing so, it brings the perspective of policy reinvention into the study of older adult sport, a field in which local policy adaptation remains underexplored. Second, it uses text similarity to measure variation in policy reinvention across provinces, thereby providing a more systematic basis for comparing how local governments redesign sports policies for older adults. Third, by combining text similarity analysis with fsQCA, it shows that high levels of policy reinvention emerge from different combinations of demographic demand, governance attention, service capacity, intergovernmental pressure, and adoption timing. These findings deepen understanding of how sports policies for older adults are translated from central directives into locally adapted policy arrangements.

## 2 Literature review and research framework

### 2.1 Research on sports policies for older adults

As population aging accelerates worldwide, the health and well-being of older adults have become central issues in public governance. The World Health Organization’s healthy aging agenda highlights the importance of sustaining health, participation, and security in later life (2). Within this broader agenda, sport and physical activity policies for older adults matter because they support functional health, reduce loneliness, and help maintain social participation (3). In the Chinese context, sports policy for older adults also serves wider governance goals by linking healthy aging to national fitness, community service provision, and the long-term reduction of health risks. Existing studies on healthy aging and sport participation support this broader policy rationale (3–5).

At the same time, sports policy research has increasingly developed its own analytical frameworks. Scholars have examined policy stages, institutional settings, advocacy processes, and sports specific policy systems, which has greatly enriched the field (8–10). However, recent reviews also show that sports policy scholarship still pays more attention to policy formation and broad implementation than to how policy texts are translated, redefined, and made operational across levels of government (11). This gap is especially important for sports policy for older adults, where implementation depends heavily on local adaptation to demographic structure, service infrastructure, and community demand. Recent reviews of sport policy research likewise note that policy content analysis remains comparatively underdeveloped relative to studies of policy formation and broad implementation (12).

Although the theoretical frameworks of policy content analysis are continuously improving, sports policies for older adults still face many challenges in practical implementation, such as ambiguous policy positioning that leads to insufficient independence (13), inefficient implementation mechanisms that weaken policy effectiveness (14), and resource supply shortages that constrain policy coverage (15). These findings imply that the key issue is not simply whether local governments adopt central policy, but how they redesign it into locally actionable provisions. Accordingly, the present study focuses on the textual reinvention of sports policy for older adults rather than on policy adoption in the abstract.

### 2.2 Research on policy reinvention

Policy reinvention refers to the extent to which adopters modify an innovation during adoption and implementation rather than merely copying it. Early policy diffusion studies were often focused on assessing whether a specific policy had been adopted in various places and the speed of that adoption as the primary goal and endpoint of research, frequently neglecting or downplaying the process of reinvention (16, 17). But later work showed that substantial content changes often occur during transmission (18–20). For policy research, this distinction matters because adoption timing and adoption content are not the same phenomenon. This conceptualization follows the classic shift from adoption centered diffusion research to reinvention-oriented analysis.

The distinction becomes especially important in vertical governance settings. In many Western countries that utilize federal systems, state governments have a relatively high level of legislative and administrative autonomy. Within the framework of federal policies, such governments can innovatively adjust policy content on the basis of factors such as the state’s financial capacity, population size, and group needs (21–23). In China, local governments operate under stronger hierarchical steering, but they still possess bounded discretion in translating central guidance into plans, implementation schemes, and operational rules (24). In this governance context, vertical reinvention refers to the phenomenon in which the central government issues policy documents, and various localities update and refine them according to their specific circumstances, which highlights the crucial role of Chinese provincial governments in the reinvention of sports policies for older adults.

Existing reinvention studies provide useful insights, but three gaps remain. First, much of the literature still emphasizes adoption or diffusion rather than the measurable degree of local reinvention in policy texts. Second, explanatory frameworks often list correlates of innovation without specifying the causal mechanism through which demand, resources, and intergovernmental relations shape reinvention. Third, the role of adoption timing remains theoretically unsettled. A longer adjustment window may enable consultation and tailoring, whereas strong administrative capacity may also allow rapid but substantive reinvention. These gaps motivate the present study. The unsettled role of timing in reinvention is especially evident in prior work on policy learning, reinvention patterns, and Chinese policy diffusion (25–27).

In short, policy reinvention is a critical concept for understanding vertical policy diffusion, but it requires sharper measurement and a more explicit causal framework. The present study addresses this need by combining text-based measurement with a configurational explanation tailored to the Chinese sports policy for older adults context.

### 2.3 Research framework

Building on diffusion research, we argue that provincial reinvention of sports policy for older adults is best understood through the interaction of four theoretical perspectives: policy capacity, policy feedback, multilevel governance, and agenda setting. Policy capacity emphasizes that governments differ in their analytical, operational, and political ability to translate broad goals into workable programs (28, 29). Policy feedback highlights how existing policies and service infrastructures reshape later policy choices by creating implementation routines, constituencies, and sunk investments (30). Multilevel governance directs attention to how central steering, local discretion, and horizontal learning jointly structure policy adaptation. Agenda-setting research further suggests that governments allocate limited attention across competing issues, and issues receiving more visible space in authoritative plans are more likely to be carried into subsequent policy design (31, 32).

In this framework, aging degree represents problem pressure. Provinces facing an aging population have a more urgent need for suitable venues, guidance, and participation opportunities for older adults, making localized policy design particularly important. Per capita provincial-level sports fiscal expenditure and public sports service resources capture complementary dimensions of sports sector policy capacity. The former reflects the fiscal support directly allocated by provincial governments to the sports field, while the latter indicates whether a province has the venue and service foundation required to implement redesigned policies. Compared to general fiscal capacity indicators, fiscal indicators in this specific area are more aligned with the field of sports policy for older adults (33, 34). This expectation is also consistent with studies on internal determinants, regional diffusion, policy learning, and comparative sports governance (22, 35–38).

The variable derived from Five-Year Plans is conceptualized here as policy salience in provincial aging planning. In agenda setting research, issue salience refers to the relative priority assigned to a problem and is commonly inferred from the share of textual attention devoted to that issue in authoritative documents (31). Recent studies likewise operationalize government attention through the textual proportions of issue-related content in reports or policy documents (64, 65). Based on this logic, the higher the proportion of content on sports for older adults in provincial aging plans, the more it indicates that sports for older adults have entered the province’s aging strategy agenda, thus increasing the likelihood that subsequent related policies will be elaborated in more detail. This interpretation is also consistent with research showing that policy attention facilitates public sector innovation and policy reinvention (32). Horizontal pressure captures interprovincial learning and competitive emulation. Provinces do not redesign policy in isolation, and they observe peer jurisdictions and may borrow, adapt, or differentiate policy clauses in response.

Finally, adoption timing is treated as a conditioning factor. Diffusion research treats adoption timing as a core temporal attribute because jurisdictions located at different points in the diffusion process face different information environments, learning opportunities, and political pressures (39, 40). Reinvention studies further suggest that early and late adopters may modify policy content for different reasons rather than along a simple monotonic line (26, 27). In some contexts, a longer adjustment window can support consultation and tailoring, while in other cases, provinces with strong policy capacity may respond quickly without resorting to textual copying. Therefore, high reinvention should emerge through multiple equifinal combinations of demand pressure, sports sector fiscal support, prior policy salience, horizontal pressure, and adoption timing. More broadly, diffusion research shows that incentives, sanctions, and multicomponent adoption processes can all shape the temporal conditions under which local redesign occurs (41, 42).

Based on the above framework, this study conceptualizes provincial policy reinvention as a process through which local governments adapt central sports policy for older adults to local demographic demand, governance attention, service capacity, intergovernmental learning, and adoption timing. The empirical analysis therefore combines text similarity with fsQCA to examine how these conditions, both individually and in combination, are associated with higher levels of policy reinvention.

## 3 Data sources and research methodology

### 3.1 Data collection

#### 3.1.1 Policy time span

The policy text sampling period for this analysis ranges from September 30, 2015, to December 31, 2024, for the following reasons:

First, the use of this period highlights a new starting point for national policies on sports for older adults. On September 30, 2015, the General Administration of Sport of China issued the “Opinions on Further Strengthening Sports Work for Older Adults under the New Situation”. This document was the first national policy on sports for older adults in China in nearly a decade, thereby filling the gap in special policies between the release of the “Notice on Strengthening Sports Work for Older Adults” in 1999 and the policy text issued in 2015. Furthermore, since 2015, the aging of China’s population has accelerated, and the demand for sports for older adults has shifted from ensuring the mere availability of basic sports facilities to emphasizing the quality and diversity of services provided. The introduction of this policy marked the official entry of sports work for older adults into a new stage of strategic advancement; hence, its release date is taken as the starting point of this research.

Second, the sample period covers the entire national strategic cycle. This period fully spans the “13th Five-Year Plan” (2016–2020) and “14th Five-Year Plan” (2021–2025) planning periods and includes the implementation stages of core strategies such as the “Healthy China 2030” planning outline and the “National Fitness Plan” (2021–2025). These strategic directions directly influence the content framework design of central and local sports policies for older adults. The complete coverage of this cycle enables a systematic exploration of the policy-strategy transmission path.

The third factor concerns policy reinvention data availability limitations. This study excludes the year 2025, primarily because of the natural lag for policy reinvention data. In accordance with the characteristics of Chinese official statistics, statistical data for the current year can be compiled and made public only by the end of the first quarter of the following year (25). Given that the policies from 2025 have not undergone a complete data statistics cycle, their key reinvention indicators cannot be accurately obtained. Thus, their inclusion could lead to analytical bias. Therefore, to ensure data completeness and validity, December 31, 2024, is set as the research endpoint.

#### 3.1.2 Policy screening method

On the one hand, the dual criteria of thematic relevance and content depth are emphasized. The study includes special policy documents on sports for older adults, such as dedicated notices, opinions, plans, and implementation schemes, as well as broader aging, health, or public-service policy documents that contain independent sections, clear targets, or concrete measures related to sports for older adults. Documents that mention sports for older adults only briefly without substantive provisions were excluded because they provide too little content for meaningful similarity analysis.

On the other hand, policy level and authority were taken into account. At the central level, the sample prioritizes documents issued by the State Council, the General Administration of Sport, the National Health Commission, and other ministries with nationwide steering authority. At the provincial level, the sample includes policy documents issued by provincial governments, autonomous regions, and municipalities directly under the central government, covering major document types such as plans, outlines, implementation opinions, and action programs. These rules improve comparability across cases and ensure that the study focuses on authoritative policy texts.

On the basis of the above screening criteria, a total of 109 policy texts on sports for older adults were collected and organized, including 6 at the central level and 103 at the local level. Some of the screening results are shown in Tables 1, 2.

**Table 1:** Screening of central government policy documents on sports for older adults.

Serial number	Publication date	Document name
1	April 13, 2022	Notice on Further Improving Sports Work for Older Adults
2	December 30, 2021	The 14th Five-Year Plan for the Development of the National Aging Cause and the Care Service System for Older Adults
3	November 18, 2021	Opinions on Strengthening Aging Work in the New Era
4	November 15, 2020	Implementation Plan on Effectively Solving the Difficulties of older adults in Using Smart Technology
5	February 28, 2017	The 13th Five-Year Plan for the Development of the National Aging Cause and the Construction of the Care System for Older Adults
6	September 30, 2015	Opinions on Further Strengthening Sports Work for older adults under the New Situation

**Table 2:** Screening results for local-level sports policies targeting older adults (Taking Fujian province as an example).

Serial number	Publication date	Document name
1	September 11, 2022	Notice from the Fujian Provincial Sports Bureau on Further Improving Sports Work for Older Adults
2	August 8, 2022	Fujian Province’s “14th Five-Year Plan” for the Development of the Care Industry for Older Adults and the Care Service System for Older Adults
3	August 7, 2022	Implementation Plan of Fujian Province for Implementing the “Opinions of the CPC Central Committee and the State Council on Strengthening Work Related to Older Adults in the New Era”
4	February 7, 2021	Notice from the Fujian Provincial Sports Bureau on Implementing the “Implementation Plan of the General Office of the State Council on Effectively Solving the Difficulties of Older Adults in Using Smart Technology”
5	July 13, 2017	Fujian Province’s “13th Five-Year Plan” for the Development of the Care Industry for Older Adults and the Construction of the Care System for Older Adults
6	March 13, 2018	Implementation Opinions of the General Office of the Fujian Provincial People’s Government on Further Strengthening Sports Work for Older Adults under the New Situation

### 3.2 Research methods

#### 3.2.1 Cosine similarity algorithm

The difference between local government policy texts on sports for older adults and central policies on sports for older adults is measured via the cosine similarity algorithm. This method has been previously used to analyze bill similarity in policy diffusion research (43). Empirical research has shown that the cosine similarity algorithm achieves moderate to high accuracy in the detection of text similarity (44). In China’s vertical policy process, a provincial policy on sports for older adults is typically issued after a central policy on the same topic and serves as its local response. Accordingly, this study applies a one-to-one document matching strategy and pairs each provincial policy text with the central policy text that corresponds most closely in topic. This procedure improves the comparability of texts and provides a clearer basis for measuring policy reinvention. The operational steps for this study were as follows:

First, text collection and preprocessing were performed. Policy documents related to sports for older adults that had been issued by the central government and 31 provincial governments were collected for the period of September 30, 2015, to December 31, 2024. First, the texts were cleaned; the titles, main body, and release dates were retained; and any irrelevant attachments and format symbols were also removed. Afterward, the precise mode of the Jieba library in conjunction with the hidden Markov model (HMM) were used for accurate word segmentation. Mainstream stop word lists, such as the Baidu Stop Words List, the Harbin Institute of Technology Stop Words List, and the Sichuan University Stop Words List, were combined to create a custom stop word removal list and filter out function words that did not carry any actual meaning.

Second, text vectorization was performed. Term frequency-inverse document frequency (TF-IDF) is a typical text analysis method. Its basic concept originates from language model theory, which involves dividing the words in a document into keywords and nonkeywords (45). Keywords are closely related to the document theme, and the importance of words can be evaluated using TF-IDF. This method compensates for the shortcomings of frequency statistics by focusing on the value of key behaviors (46). In this study, the TF-IDF algorithm is used to convert policy texts into numerical vectors. The formula is as follows:


$$\mathrm{TF}-\mathrm{IDF}(t,d)=\mathrm{TF}(t,d)\times \log(\frac{N}{\mathrm{DF}(t)+1})$$

where “t” is the term, “d” is the document, N is the total number of documents, and DF(“t”) is the number of documents containing “t.”

Finally, a similarity calculation is performed. Before cosine similarity was calculated, the TF-IDF vectors were L2-normalized. This procedure helps reduce the mechanical influence of document length on similarity scores and makes the comparison focus more on differences in substantive term distributions rather than on absolute text size. The cosine similarity between the central policy term frequency vector A and the local policy term frequency vector B is calculated as follows:


$$\text{cosSim}(A,B)=\frac{A\cdot B}{|A|\times |B|}=\frac{\sum_{i=1}^{n}A_{i}\times B_{i}}{\sqrt{\sum_{i=1}^{n}A_{i}^{2}}\times \sqrt{\sum_{i=1}^{n}B_{i}^{2}}}$$

In this study, the reinvention coefficient is defined as 1 minus the similarity between a specific policy text issued by the central government and the corresponding text issued by a province. Its value ranges from 0 to 1. A higher value indicates a lower similarity between the local government’s and central government’s policy texts. That is, the local government has made a greater degree of localized modifications to the policy content. On the basis of the concept of policy reinvention that was discussed earlier, a higher coefficient value is equivalent to a higher degree of policy reinvention by the local government.

#### 3.2.2 Fuzzy-set qualitative comparative analysis (fsQCA)

To explain why reinvention varies across provinces and planning stages, this study uses fsQCA to identify multiple conjunctural paths to high reinvention. The analysis is conducted separately for the 13th Five-Year Plan stage (2016–2020) and the observed 14th Five-Year Plan stage (2021–2024), following the strategy of conducting multiple QCAs across different time periods (47). The unit of analysis is the province within each planning stage. The outcome is the stage-specific provincial reinvention coefficient, calculated as the average reinvention score of the valid policy texts on sports for older adults issued by a province in the corresponding stage. Six antecedent conditions are examined: per capita provincial-level sports fiscal expenditure, aging degree, public sports service resources, policy salience in aging planning, horizontal pressure, and adoption timing. fsQCA is well suited to this task because it can identify multiple conjunctural paths and has been increasingly used in sports policy research (48–51).

Because the outcome depends on the existence of valid local policy texts, provinces without a valid policy text in a given planning stage are not assigned artificial reinvention scores and are excluded from that stage-specific fsQCA. Guangdong is included only in the observed 14th Five-Year Plan stage, while Heilongjiang, Hubei, Liaoning, Shaanxi, and Tibet are included only in the 13th Five-Year Plan stage. Provinces with valid policy texts in both stages enter both period-specific analyses. This treatment preserves the textual basis of the reinvention coefficient and avoids imputing policy redesign where no local policy text is observed.

This periodized case strategy also clarifies the scope of comparison. Each stage-specific fsQCA examines the observed provincial cases within that planning period, while the full 2015–2024 policy corpus is retained for descriptive measurement of overall policy reinvention. This approach addresses temporal heterogeneity without forcing provinces into a stage for which no local policy text exists.

The operational measurement of the antecedent conditions is explained as follows:

Per capita provincial-level sports fiscal expenditure. This condition captures the sports-sector fiscal resources directly allocated by provincial governments to support policy design and implementation in the field of sport. Because sports policies for older adults require financial support for venue adaptation, service provision, activity organization, and cross-departmental implementation, a sports-specific fiscal indicator is more closely aligned with the policy domain than a general fiscal-capacity measure (33, 34, 66). Specifically, provincial-level sports fiscal expenditure from the general public budget is divided by the resident population of each province. For the periodized analysis, the average value is calculated separately for 2016–2020 and 2021–2024, and the stage-specific value is used in the corresponding fsQCA model.

##### 3.2.2.1 Aging degree

The degree of aging directly reflects the intensity of local demand for sports policies for older adults. A higher demand intensity provides a stronger driving force for the introduction and improvement of relevant policies (52). Specifically, the proportion of the population aged 65 and above in the total resident population of each province is calculated separately for 2016–2020 and 2021–2024. The stage-specific average is used in the corresponding periodized fsQCA model.

##### 3.2.2.2 Public sports service resources

Public sports service resources are the basic conditions under which local governments provide sports services for older adults (3). This condition is measured by per capita sports venue area. To align the antecedent conditions with the periodized outcome, the average value is calculated separately for 2016–2020 and 2021–2024 and then used in the corresponding stage-specific analysis.

##### 3.2.2.3 Policy salience in aging planning

The proportion of content on sports for older adults in provincial 13th and 14th Five-Year Plans for the development of aging undertakings and care service system for older adults is used as a proxy for the strategic salience of sports for older adults in the provincial policy agenda. This operationalization follows agenda setting studies that infer issue salience from the share of attention devoted to an issue in authoritative texts (31). The average value is calculated separately for 2016–2020 and 2021–2024 and then used in the corresponding stage-specific analysis.

##### 3.2.2.4 Horizontal pressure

Horizontal pressure reflects the demonstration and competition effects of peer governments on local policy reinvention. When the surrounding or peer regions successively issue sports policies for older adults, a learning or competition effect is created (53). In the periodized analysis, this condition is calculated within the corresponding planning stage by identifying the number of other provinces that had already issued similar policy texts before a focal provincial policy was formulated. When a province issued more than one valid policy text in the same stage, the average value is used.

##### 3.2.2.5 Adoption timing

Following diffusion studies that operationalize adoption speed or timing as the interval or temporal position of adoption within a diffusion sequence (39, 40), this study uses the interval between the release of a central policy and the release of the corresponding provincial policy as a timing condition. In the periodized analysis, adoption timing is calculated only for valid local policy texts issued in the corresponding stage. When a province issued more than one valid policy text in the same stage, the average interval is used.

## 4 Results and analysis

### 4.1 Measurement of the policy reinvention coefficient

To explore the factors and mechanisms influencing local policy reinvention, this study first aimed to measure the degree of reinvention in sports policies for older adults across provinces. This was achieved by calculating a policy reinvention coefficient based on text similarity analysis between central and local policy documents. The calculation results are shown in Table 3.

**Table 3:** Descriptive statistics related to local government reinvention coefficients of sports policies for older adults.

Province	Average	Maximum	Minimum
Anhui	0.6314	0.7435	0.4406
Beijing	0.8445	0.8934	0.7283
Chongqing	0.7919	0.8615	0.7440
Fujian	0.6413	0.8468	0.4714
Gansu	0.7807	0.8520	0.7094
Guangdong	0.6037	0.6037	0.6037
Guangxi	0.6670	0.7719	0.5839
Guizhou	0.7079	0.8848	0.5777
Hainan	0.7877	0.8400	0.7298
Hebei	0.7345	0.8475	0.5806
Heilongjiang	0.6059	0.6059	0.6059
Henan	0.5951	0.8978	0.2436
Hubei	0.8360	0.8360	0.8360
Hunan	0.6794	0.8276	0.5741
Jiangsu	0.7370	0.9092	0.5606
Jiangxi	0.6004	0.7848	0.3942
Jilin	0.6549	0.7698	0.5276
Liaoning	0.8306	0.8306	0.8306
Inner Mongolia	0.8171	0.9121	0.7221
Ningxia	0.4901	0.5796	0.4006
Qinghai	0.7201	0.9217	0.4023
Shandong	0.7231	0.8356	0.6704
Shanghai	0.7997	0.9043	0.6018
Shanxi	0.6573	0.8521	0.4059
Shaanxi	0.8055	0.8055	0.8055
Sichuan	0.7599	0.9075	0.6035
Tianjin	0.8004	0.9158	0.6782
Xinjiang	0.6315	0.8112	0.3480
Tibet	0.5891	0.5891	0.5891
Yunnan	0.6662	0.8700	0.5482
Zhejiang	0.7998	0.9282	0.6634
Total	0.7093		

Among the 31 provincial-level administrative units, only one valid policy text was retrieved for 6 provincial governments: Guangdong, Heilongjiang, Hubei, Liaoning, Shaanxi, and Tibet. Since a single text cannot reflect the stability and overall characteristics of policy reinvention through average calculations, such calculations were excluded from the reinvention degree ranking. The final sample used in ranking analysis included 25 provincial governments. By calculating the reinvention coefficient, among these 25 provincial local governments, Beijing has the highest degree of policy reinvention, with an average reinvention coefficient for its multiple sports policies of 0.8445. The local government with the lowest degree of reinvention is the Ningxia Hui Autonomous Region, whose average reinvention coefficient for its multiple sports policies is 0.4901. The overall average policy reinvention coefficient among local governments is 0.7093, indicating a relatively high overall level of reinvention of sports policies for older adults, and the core requirements of central policies are innovatively transmitted and implemented at the local level.

In short, the text similarity analysis reveals meaningful provincial variation but also a relatively high overall level of reinvention. This finding establishes the empirical foundation for asking why some provinces redesign central policy more extensively than others.

### 4.2 Temporal and spatial differences in reinvention of sports policies for older adults

Having established the reinvention coefficient, this section examines its temporal and spatial distribution. The purpose is not merely descriptive. Patterns over time and across regions help identify whether reinvention is episodic, convergent, or structurally uneven before the configurational analysis turns to causal explanation.

**Figure 1:**
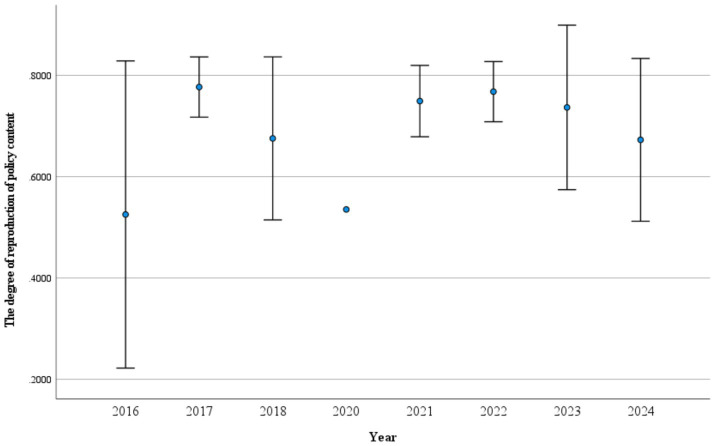
Differences in policy reinvention among sports policies for older adults across different years of publication. The blue circles represent the mean value of policy content reinvention, and the black lines denote the 95% confidence interval.

Figure 1 presents the annual evolution of the provincial reinvention coefficients of sports policies for older adults from 2015 to 2024. The coefficient fluctuated more strongly between 2016 and 2020, then became more stable after 2020. This pattern suggests a process in which provinces moved from initial adjustment to a more mature phase of policy translation. As sports for older adults became more firmly embedded in the healthy aging and national fitness agendas, local governments appeared to develop a more settled repertoire for adapting central directives.

**Figure 2:**
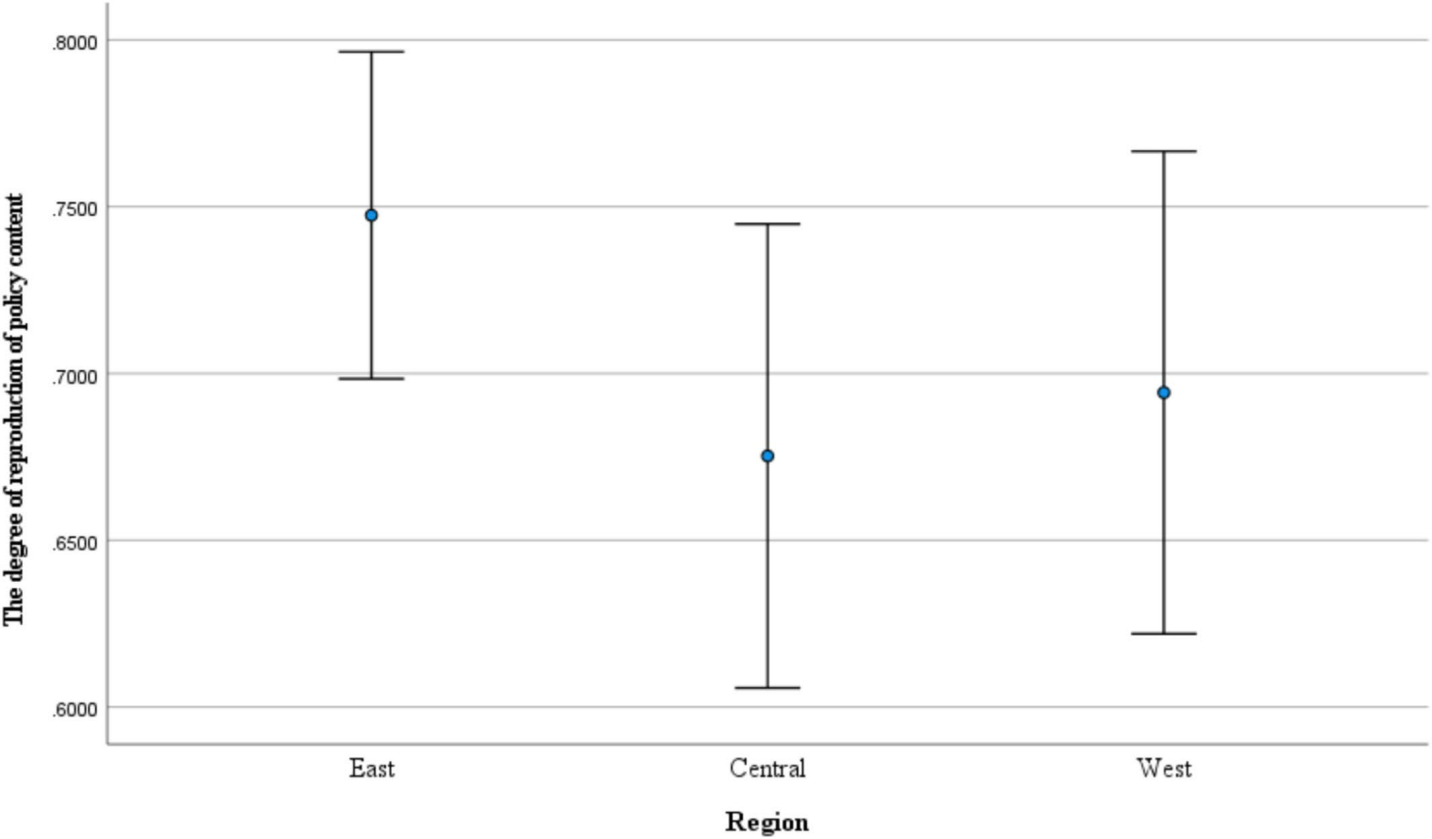
Variations in the degree of reinvention of sports policies for older adults across different regions. The blue circles represent the mean value of policy content reinvention, and the black lines denote the 95% confidence interval.

In the spatial difference analysis, on the basis of China’s national development plan, the provincial local governments are divided into eastern, central, and western regions. The degree of policy content in sports policies for older adults is separately calculated in different regions. As shown in Figure 2, the eastern region has the highest average reinvention coefficients of sports policies content for older adults. The central and western regions have similar averages, thereby showing convergent characteristics. Although the economic base and aging service resources differ across the three regions (east, central, and west), overall, the degree of policy content reinvention is relatively high in all three regions, with small differences in their average values.

Therefore, the analysis of temporal and spatial differences demonstrates that policy reinvention has stabilized over time after an initial period of adjustment and shows a pattern of regional convergence, indicating a maturing and standardized approach to local policy adaptation across China.

The previous sections clearly present the temporal and spatial differences in the degree of reinvention of sports policies for older adults through quantitative analysis. However, this descriptive analysis shows how reinvention varies, but not how multiple conditions work together to produce high reinvention. Because provincial policy reinvention is unlikely to be driven by a single net effect, a configurational approach is needed to examine how demographic demand, sports sector fiscal support, public sports service resources, planning salience, horizontal pressure, and adoption timing jointly shape reinvention during the 13th and 14th Five-Year Plan stages.

### 4.3 Conditional configuration analysis of policy content reinvention in sports policies for older adults

#### 4.3.1 Variable calibration

Before conducting the necessary-condition and sufficiency analyses, the outcome and antecedent conditions must be calibrated into fuzzy-set membership scores. In this study, direct calibration was used because there are no universally accepted external thresholds for defining high or low levels of provincial policy reinvention, per capita provincial-level sports fiscal expenditure, planning salience, or adoption timing in the context of sports policy for older adults. For the periodized analysis, calibration is conducted separately within each stage-specific case set, because the observed cases and empirical distributions may differ between the 13th and 14th Five-Year Plan stages.

More specifically, the raw anchors for full-set membership, the crossover point, and full-set non-membership were set with reference to the upper quartile, median, and lower quartile of each variable’s empirical distribution within the corresponding stage-specific case set. These three substantive anchor values were then mapped onto the conventional fuzzy-set thresholds of 0.95, 0.50, and 0.05 through the direct calibration procedure (54, 55). This approach retains consistency with established fsQCA practice while grounding calibration in the distributional characteristics of the present dataset. The calibration results are reported in Tables 4, 5.

**Table 4:** Variable calibration results for the 13th Five-Year Plan stage.

Variable type	Variable name	Calibration anchor point		
		Full-set membership	Intermediate-set membership	Full-set nonmembership
Result Variable	Reinvention coefficient of sports policies for older adults	0.8499	0.7194	0.5222
Condition Variable	Per capita provincial-level sports fiscal expenditure	87.3351	7.4628	3.6579
	Aging degree	15.1188	11.8630	7.7500
	Public sports service resources	2.5962	1.7940	1.2887
	Policy salience in aging planning	0.0325	0.0201	0.0099
	Horizontal pressure	17.8250	9.2500	1.4500
	Policy adoption timing	0.9271	0.3959	0.1590

**Table 5:** Variable calibration results for the 14th Five-Year Plan stage.

Variable type	Variable name	Calibration anchor point		
		Full-set membership	Intermediate-set membership	Full-set nonmembership
Result variable	Reinvention coefficient of sports policies for older adults	0.9149	0.7156	0.6005
Condition variable	Per capita provincial-level sports fiscal expenditure	66.2923	8.6642	3.9288
	Aging degree	18.3781	14.8663	9.7525
	Public sports service resources	3.8363	2.6600	2.3350
	Policy salience in aging planning	0.0274	0.0142	0.0053
	Horizontal pressure	13.7500	5.5000	1.0000
	Policy adoption timing	1.8418	0.6046	0.1219

Precise values of 0 and 1 were not used because they correspond to negative and positive infinity in the log-odds transformation and would therefore create computational problems in fuzzy-set analysis (55). In addition, when a case was calibrated exactly at the crossover point of 0.50, a constant of 0.001 was added to distinguish set membership more clearly and to avoid ambiguity in subsequent configuration analysis (56). Through this procedure, all outcome and condition variables were transformed into fuzzy-set scores ranging from 0 to 1 and made suitable for the subsequent necessary-condition and configurational analyses.

#### 4.3.2 Analysis of necessary conditions

Prior to configuration analysis, a necessary condition analysis for single variables that uses consistency as the main reference indicator needs to be conducted. The consistency threshold for necessary analysis is typically set at 0.9, which means that if a certain condition is universal for the achievement of the outcome variable, its consistency must reach or exceed 0.9 (57). Specifically, fsQCA4.1 software is used to test the consistency and coverage of all the condition variables. The specific results are shown in Tables 6, 7.

**Table 6:** Necessary condition analysis results for the 13th Five-Year Plan stage.

Condition variable	High reinvention coefficient of sports policies for older adults	
	Consistency	Coverage
Per capita provincial-level sports fiscal expenditure	0.640494	0.773526
~ Per capita provincial-level sports fiscal expenditure	0.635023	0.541221
Aging degree	0.753836	0.737117
~Aging degree	0.490994	0.501704
Public sports service resources	0.703803	0.690445
~Public sports service resources	0.557705	0.567935
Policy salience in aging planning	0.695130	0.662428
~Policy salience in aging planning	0.557038	0.585144
Horizontal pressure	0.589727	0.620787
~Horizontal pressure	0.659106	0.626904
Policy adoption timing	0.498999	0.541636
~Policy adoption timing	0.770514	0.713403

~denotes logical negation, indicating that the variable is at a lower level.

**Table 7:** Necessary condition analysis results for the 14th Five-Year Plan stage.

Condition variable	High reinvention coefficient of sports policies for older adults	
	Consistency	Coverage
Per capita provincial-level sports fiscal expenditure	0.567839	0.740637
~ Per capita provincial-level sports fiscal expenditure	0.677674	0.616188
Aging degree	0.718593	0.775969
~Aging degree	0.529792	0.563359
Public sports service resources	0.610912	0.750441
~Public sports service resources	0.650395	0.618008
Policy salience in aging planning	0.636037	0.703733
~Policy salience in aging planning	0.638191	0.662938
Horizontal pressure	0.655420	0.725179
~Horizontal pressure	0.590811	0.613721
Policy adoption timing	0.638981	0.705141
~Policy adoption timing	0.643862	0.670479

~denotes logical negation, indicating that the variable is at a lower level.

The test results reveal that the consistency of each antecedent condition examined in this study is less than the necessary-condition critical value of 0.9. This finding indicates that high reinvention of sports policies for older adults does not depend on any single factor operating on its own. Instead, reinvention is best understood as the outcome of combinations of demand pressure, per capita provincial-level sports fiscal expenditure, planning salience, horizontal pressure, and adoption timing. This is precisely the type of causal complexity that fsQCA is designed to identify.

#### 4.3.3 Configuration path analysis

When a configuration analysis is conducted, it is necessary to set the raw consistency threshold, frequency threshold, and PRI consistency value according to the research context. First, the raw consistency threshold is determined. Related studies generally believe that the minimum value of this threshold should not be lower than 0.75, and many scholars use 0.8 as the benchmark reference value (58). Combined with the natural breakpoints of the consistency values used in this study, the raw consistency threshold is ultimately set at 0.8. Second, the frequency threshold was set to 1 because each period-specific model uses a small-N case set and aims to preserve all observed provincial cases in the corresponding planning stage. Third, the PRI consistency threshold is determined. Usually, a high PRI that is close to the raw consistency score is desired. Configurations with PRI scores of less than 0.5 indicate significant inconsistency (59). In combination with the data characteristics of this study, the PRI value is ultimately determined to be 0.7. Finally, for the results presentation method, the QCA analysis results reporting framework proposed by Fiss (54) is used for reference. The paper reports the intermediate solution, because it balances theoretical guidance and empirical conservatism and is the standard basis for distinguishing core and peripheral conditions. Core conditions are identified by comparing the intermediate and parsimonious solutions. The directional expectations for high reinvention are positive for per capita provincial-level sports fiscal expenditure, aging degree, public sports service resources, policy salience, and horizontal pressure. For adoption timing, no single directional expectation is imposed because both prompt response and longer adjustment windows are theoretically plausible depending on the surrounding conditions. The specific configuration results are shown in Tables 8, 9.

**Table 8:** Configuration analysis results for the 13th Five-Year Plan stage.

Prerequisite Conditions	Delayed learning-adjustment type			Efficiency-Optimal Type	Constraint-compensating type
	Path 1	Path 2	Path 3	Path 4	Path 5
Per capita provincial-level sports fiscal expenditure	●	●		●	⊗
Aging degree		⊗	●	●	●
Public sports service resources	●		●		⊗
Policy salience in aging planning		●	●	⊗	⊗
Horizontal pressure	⊗	⊗	⊗	●	●
Policy adoption timing	⊗	⊗	⊗	●	⊗
Consistency	0.978	0.980	0.904	0.972	0.903
Raw coverage	0.453	0.288	0.394	0.258	0.254
Unique coverage	0.062	0.036	0.055	0.044	0.041
Total coverage	0.697				
Total consistency	0.904				

Black circles (●) indicate the presence of a condition, and circles with an “x”(⊗) indicate their absence. Large circles indicate core condition, and small circles indicate peripheral condition; “blank” sections indicate conditions that may either be present or absent.

**Table 9:** Configuration analysis results for the 14th Five-Year Plan stage.

Prerequisite Conditions	Fiscal-demand type			Horizontal-learning type		Aging-response type	
	Path 1	Path 2	Path 3	Path 4	Path 5	Path 6	Path 7
Per capita provincial-level sports fiscal expenditure	●	●	●	⊗	⊗	⊗	⊗
Aging degree	●	●	●	●	●	●	●
Public sports service resources		⊗	●	●		●	⊗
Policy salience in aging planning	⊗		●		●	⊗	⊗
Horizontal pressure	⊗	⊗	●	●	●	⊗	●
Policy adoption timing	⊗	⊗	●	⊗	⊗	●	●
Consistency	0.886	0.889	0.985	0.980	0.956	0.952	0.939
Raw coverage	0.279	0.289	0.241	0.276	0.267	0.215	0.277
Unique coverage	0.019	0.024	0.030	0.022	0.022	0.019	0.065
Total coverage	0.614						
Total consistency	0.928						

Black circles (●) indicate the presence of a condition, and circles with an “x”(⊗) indicate their absence. Large circles indicate core condition, and small circles indicate peripheral condition; “blank” sections indicate conditions that may either be present or absent.

Tables 8, 9 show that all stage-specific configurations reach acceptable consistency levels. The 13th Five-Year Plan model identifies five paths, with an overall solution consistency of 0.904 and overall coverage of 0.697. The observed 14th Five-Year Plan model identifies seven paths, with an overall solution consistency of 0.928 and overall coverage of 0.614. These results indicate that policy reinvention follows different mechanisms across planning stages. The 13th Five-Year Plan stage reflects an earlier process of policy learning and local adjustment, while the observed 14th Five-Year Plan stage shows a stronger connection between aging pressure, sports sector fiscal support, and horizontal learning.

1. *The 13th Five-Year Plan stage*: The five paths in this stage can be grouped into three types. The first is the delayed learning-adjustment type, including Paths 1, 2, and 3. These paths are generally characterized by slower adoption, which means a longer interval between central policy release and provincial response. This pattern suggests that, in the early stage of sports policy for older adults, some provinces needed more time to interpret central policy signals, assess local service conditions, and translate general requirements into feasible local provisions. Path 1 relies on sports fiscal support and public sports service resources, Path 2 relies on sports fiscal support and policy salience, and Path 3 relies on aging pressure and policy salience. Although their specific combinations differ, all three paths show that delayed adoption can create room for policy learning when it is supported by fiscal, service, or agenda conditions. Hubei is a representative case in this stage. Public reports describe Hubei as having expanded sports facilities for people of different age groups in both urban and rural areas, which helps explain why service resources could support more detailed local policy redesign.

The second type is the rapid capacity-response type, represented by Path 4. This path combines high sports fiscal expenditure and high aging degree as core conditions, with faster adoption and horizontal pressure as peripheral supports. Its mechanism lies in the ability of some provinces to convert fiscal resources and demographic demand into rapid policy response. In such cases, faster adoption does not necessarily imply simple copying. It may indicate that the province already had the fiscal and administrative basis needed to refine policy content within a shorter response period.

The third type is the constraint-compensating type, represented by Path 5. This path combines weaker sports fiscal support with slower adoption and horizontal pressure. Its mechanism is compensatory. When fiscal input is limited, provinces may still achieve policy reinvention by extending the adjustment period, observing peer experience, and reorganizing existing service arrangements. This type shows that policy learning and external pressure can partly offset weaker sports sector fiscal support during the early diffusion stage.

1. *The 14th Five-Year Plan stage*: The seven paths in this stage can also be grouped into three types. The first is the fiscal-demand type, including Paths 1, 2, and 5. These paths are centered on high sports fiscal expenditure and high aging degree. This indicates that, as sports for older adults became more closely connected with healthy aging and national fitness agendas, local reinvention became more dependent on the joint effect of demographic pressure and sports sector fiscal support. Path 5 also includes public sports service resources, policy salience, faster adoption, and horizontal pressure as peripheral conditions, showing a more complete governance support structure. Zhejiang is a useful example. In 2022, Zhejiang upgraded its“Zhejiang Fitness” service and included an “Sports Service for Older Adults” section that recommends activities such as Taijiquan, Health Qigong, Rouliqiu, and square dance according to fitness needs of older adults.

The second type is the horizontal-learning type, including Paths 3 and 4. These paths are characterized by slower adoption and stronger horizontal pressure as core conditions. Their mechanism is interprovincial learning. By the 14th Five-Year Plan stage, more provinces had accumulated policy experience, which provided late-responding provinces with clearer reference points. A longer response interval in this stage may therefore reflect policy comparison, selective borrowing, and local refinement.

The third type is the aging-response type, including Paths 6 and 7. These paths combine high aging degree, low policy salience in aging planning, and faster adoption. This pattern suggests that some provinces responded directly to demographic pressure even when sports for older adults did not occupy a highly visible position in aging plans. Public sports service resources or horizontal pressure then provided additional support for redesign. This type reflects a more practical response to aging-related service demand.

1. *Cross-stage comparison*: The periodized results show both continuity and change. Across both stages, high reinvention is produced by combinations of demand, fiscal support, service resources, policy attention, adoption timing, and horizontal pressure. The main difference lies in the dominant mechanism. During the 13th Five-Year Plan stage, slower adoption appears repeatedly, suggesting that local governments often relied on time for learning, resource preparation, and policy translation. During the observed 14th Five-Year Plan stage, aging degree appears in all seven paths, and sports fiscal support becomes central in several paths. This shift suggests that, as the policy field matured, local reinvention became more closely tied to demographic pressure and domain-specific fiscal input.

The role of public sports service resources also becomes more conditional in the periodized results. In the earlier stage, service resources often served as the basic carrier for policy redesign. In the later stage, they more often worked together with aging pressure, fiscal support, or horizontal learning. This change indicates that local policy reinvention gradually moved from basic adaptation to more differentiated redesign shaped by demand pressure and governance capacity.

#### 4.3.4 Robustness test

The fsQCA results may be affected by calibration choices and parameter settings, so robustness checks remain necessary for the periodized models. Following established practice in fsQCA robustness assessment (60), this study applies the same parameter-adjustment strategy to each planning-stage model. Specifically, the raw consistency and PRI consistency thresholds are increased under a more conservative specification, while the frequency threshold remains at 1 because each model retains the observed provincial cases within the corresponding stage.

The robustness test is interpreted as a threshold-based stability check for the periodized configurations. The key issue is whether the main stage-specific mechanism types remain substantively stable after stricter thresholds are applied. This procedure helps assess whether the identified 13th and 14th Five-Year Plan configurations are driven by a single parameter choice or reflect more stable patterns of provincial policy reinvention.

## 5 Conclusion

### 5.1 Conclusions and discussion

This study aimed to measure and explain the variation in how Chinese provincial governments adapt national sports policies for older adults. By analyzing policy texts and applying a configurational approach, the research provides insights into the complex mechanisms of local policy reinvention.

1. The overall level of reinvention of sports policies for older adults is relatively high, with significant interprovincial differences but without extreme polarization. The average reinvention coefficient indicates that local governments are not merely copying central directives but are *actively* engaging in adaptation. Similar to Chen and Huang (25), this finding suggests a generally effective vertical policy transmission system within China’s governance model. Differences between higher- and lower-reinvention provinces indicate that local adaptation is shaped by a combination of demographic demand, sports sector fiscal support, policy attention, and service resources.
2. Reinvention of sports policies for older adults is characterized by temporal stabilization and spatial convergence. In terms of time, the policy reinvention coefficient showed an evolutionary trend of fluctuation followed by stability over the period of 2015 to 2024. The shift from initial fluctuation post-2015 to stable adaptation after 2020 indicates that local policy processes have matured into a standardized paradigm. This aligns with the concept of policy learning curves described in literature (38, 61). In terms of space, with its reliance on economic and public service advantages, the eastern region has an average coefficient of close to 0.75, which is higher than those of the central and western regions. The average coefficients of the central and western provinces are both close to 0.70, with small regional differences. This differs from the findings in other public service literature, which conclude that there are significant variations in the regions where policy reinvention occurs (6, 62).
3. A core theoretical contribution is the confirmation that high reinvention is not driven by a single factor but results from the conjunction of multiple conditions, supporting complex causality perspectives in policy studies. The periodized results strengthen this point by showing that different combinations work in different planning stages. In the 13th Five-Year Plan stage, high reinvention was mainly generated through delayed learning-adjustment, rapid capacity-response, and constraint-compensating paths. In the observed 14th Five-Year Plan stage, high reinvention was mainly generated through fiscal-demand, horizontal-learning, and aging-response paths. This finding extends prior diffusion and reinvention research by showing that the temporal context of policy transmission shapes how local governments redesign central policy (26, 27, 47).
4. This study provides evidence that domain-specific fiscal support matters for the reinvention of sports policies for older adults. The results show that sports fiscal support is especially important when combined with aging pressure, service resources, or rapid policy response. This confirms that local governments require not only general governing capacity but also sports-sector resources that can be converted into age-friendly venues, activity programs, digital services, and implementation support.

The existing research on policy innovation is often focused on the diffusion of a single policy across regions [Glick and Hays,1991; Hays,1996; (63)]. In contrast, our study examines the diffusion and innovation of six policies related to sports for older adults over time. Like that of Jansa et al. (61), our research offers empirical support that explains how differences among local governments lead to varying levels of policy reinvention. The periodized analysis further shows that the mechanisms of reinvention change over time, which supports the methodological value of tracking configurations across different periods (47).

### 5.2 Suggestions for policy implementation

1. Interprovincial pairing assistance should be promoted to narrow the gap in the degree of policy reinvention. Those provinces with high degrees of reinvention, such as Beijing, Jiangsu, and Zhejiang, should be encouraged to share their innovation experiences in age-friendly service refinement, digital platform construction, etc., with provinces such as Ningxia, Tibet, and Henan through policy text sharing and experience-exchange meetings. Lagging provinces need to focus on their own shortcomings. For example, Ningxia could strengthen local policy adaptation by adding specific provisions for ethnic and folk sports activities for older adults, with attention to the exercise preferences of Hui older adults.
2. Localities should be guided to choose policy reinvention strategies according to their resource endowments and planning stage conditions. Provinces with stronger sports fiscal support and higher aging pressure should use fiscal resources to refine age-friendly facility standards, community activity provision, and digital service access. Provinces with weaker sports fiscal support should focus on reorganizing existing venue networks, strengthening horizontal learning, and improving the local fit of policy clauses. Since the 13th and 14th Five-Year Plan stages show different mechanisms, provincial governments should avoid a uniform model and instead align policy redesign with their fiscal resources, aging demand, service base, and timing of policy response.
3. A multidepartment coordination mechanism needs to be constructed to support the joint operation of the core conditions identified in different planning stages. Since per capita provincial-level sports fiscal expenditure, aging degree, public sports service resources, policy salience in aging planning, adoption timing, and horizontal pressure play different roles across configurations, provincial governments should strengthen coordination among sports, finance, health, civil affairs, and community governance departments. Such coordination can improve the conversion of sports fiscal resources and service platforms into concrete policy measures for older adults. Professional institutions can also be introduced through government purchase of services to provide training for older-adult sport instructors and to improve the implementation quality of locally redesigned policies.
4. The long-term guarantee systems should be improved to consolidate the stability of policy reinvention. A long-term mechanism is built from three aspects: evaluation, feedback, and iteration. Third-party institutions should regularly evaluate the effectiveness of reinvention in sports policies for older adults, covering dimensions such as degree of policy refinement and implementation conversion rate. At the feedback level, on the basis of community grid members and sports associations for older adults, feedback channels for policy implementation problems need to be established to promptly address issues of insufficient adaptation during policy execution. At the iteration level, local governments need to regularly and dynamically optimize sports policies for older adults on the basis of evaluation results and feedback, thereby ensuring that policy content remains synchronized with the needs of the older population and the stage of social development.

### 5.3 Limitations

This study has several limitations. First, the measurement of policy reinvention is based on a clearly specified text-similarity approach and a central–provincial one-to-one matching strategy, but the use of a single baseline metric means that the results should be interpreted with appropriate caution. Second, although the periodized design reduces the risk that full-period averages smooth over planning stage differences, the number of valid policy texts differs across the 13th and 14th Five-Year Plan stages. This may affect the stability of some stage-specific configurations. Third, the analysis covers observed provincial cases within each planning stage, so provinces without valid policy texts in a given stage are excluded from that stage-specific fsQCA. This preserves the textual basis of the outcome variable but limits direct comparison between the two stage-specific case sets. Finally, the study focuses on a limited set of theoretically relevant structural conditions, while other contextual factors may also shape how central sports policies for older adults are translated into local policy design. Future research could further refine the measurement and extend the explanatory scope of this line of inquiry.

## Data Availability

The original contributions presented in the study are included in the article/Supplementary material, further inquiries can be directed to the corresponding author.
